# Ion and Site Correlations of Charge Regulating Surfaces: A Simple and Accurate Theory

**DOI:** 10.1021/acs.langmuir.3c00316

**Published:** 2023-05-24

**Authors:** Martin Trulsson

**Affiliations:** Computational Chemistry, Lund University, Lund SE-221 00, Sweden

## Abstract

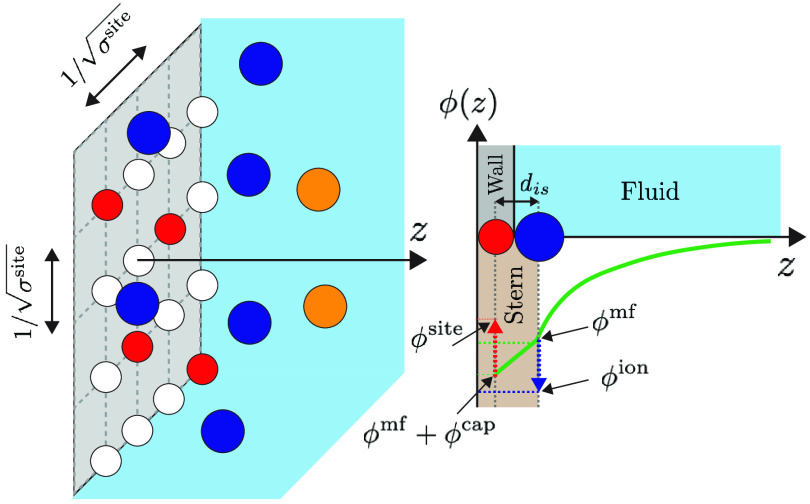

Charge regulation is fundamental in most chemical, geochemical, and biochemical systems. Various mineral surfaces and proteins are well-known to change their charge state as a function of the activity of the hydronium ions, that is, the pH. Besides being modulated by the pH, the charge state is sensitive to salt concentration and composition due to screening and ion correlations. Given the importance of electrostatic interactions, a reliable and straightforward theory of charge regulation would be of utmost importance. This Article presents a theory that accounts for salt screening, site, and ion correlations. Our approach shows an impeccable agreement as compared to Monte Carlo simulations and experiments of 1:1 and 2:1 salts. We furthermore disentangle the relative importance of site–site, ion–ion, and ion–site correlations. Contrary to previous claims, we find that ion–site correlations for the studied cases are subdominant to the two other correlation terms.

## Introduction

Surface dissociation or association of hydronium ions in aqueous solutions is very important for many chemical and biological systems. Immersed nanoparticles (e.g., proteins^1^) or surfaces^2^ acquire electrostatic charges regulated by hydronium ion concentration, salt concentration, and many other factors. The electrostatic charge is one key factor for stabilizing colloidal suspensions, as they would otherwise aggregate due to attractive van der Waals forces unless other protective interactions are at play (e.g., sterically repelling polymer brushes). The praised DLVO-theory,^3,4^ including attractive van der Waals forces and repulsive electrostatic forces, helps us to predict colloidal stability between equal and simple colloidal particles. It successfully predicts a lowered stability at higher salt concentrations as stabilizing electrostatic forces are screened out.

The most straightforward approach to predict a surface’s charge, as needed for the DLVO theory, uses the Henderson–Hasselbalch formula,^5^ which relates the surface ionization to the hydronium ion concentration. However, this formula does not account for the electrostatic potential buildup at the surface due to the mutual electrostatic energy between the charged surface and its diffuse counterion layer. Hence, it typically overestimates the surface charge density. To account for this effect, one typically relies on Poisson–Boltzmann calculations^6−8^ or Monte Carlo (MC) simulations,^9,10^ both of which account for both pH and salt concentration dependence.^10^ Unlike MC simulations, the Poisson–Boltzmann theory is well-known to be insufficient as soon as the counterions of the charged surface are multivalent (divalent and above) due to a lack of ion–ion correlations in the mean-field theory. Moreover, Poisson–Boltzmann calculations generally do not account for the discreteness of the surface sites, even though Bakhshandeh et al.^11^ have recently accounted for the latter by showing an excellent agreement between theory and MC simulations of charge regulating nanoparticles in the presence of 1:1 salts.

Here, we extend this work, accounting for ion correlations, with a straightforward yet highly accurate theory for planar surfaces. We compare our theoretical predictions to available experimental data^2^ and previous MC simulations,^10^ within the primitive model, both in 1:1 and in 2:1 salt solutions and with excellent agreement. Moreover, we disentangle the various contributions to the overall titration behavior and find that site–site correlations are important for all of the studied cases. Ion–ion correlations are, as expected, only relevant at high electrostatic coupling parameters, that is, when the excess counterion concentration is high close to the surface due to a high surface charge density and when these counterions are multivalent. In contrast to previous claims,^10^ we find that the ion–site correlations are generally subdominant.

## Methods and Theory

Instead of solving the full nonlinear Poisson–Boltzmann equation, we rely on just solving the boundary condition, assuming a flat impenetrable titratable surface. The (surface charge modulated) contact theorem^12−15^ specifies that
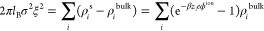
1where *l*_B_ is the Bjerrum length, σ is the surface charge density (in inverse area units), *e* is the elementary charge, ρ_*i*_^bulk^ and ρ_*i*_^s^ are the bulk reservoir and surface densities of species *i*, *z*_*i*_ is the valency of species *i*, β ≡ 1/*k*_B_*T* is the inverse temperature, and ϕ^ion^ is the electrostatic potential of an ion sitting on the surface. In eq 1, we introduced a ξ^2^ ≥ 1 parameter, which is not normally included in the contact theorem. This parameter accounts for ion–site correlations, which give rise to additional forces over the interface as compared to when surface sites are uniformly smeared-out. These extra correlations occur when surfaces bear discrete charges and when the electrostatic couplings start to be important^16^ but also appear within the Poisson–Boltzmann theory (low-coupling regime) for charge-modulated surfaces.^17^ For uniformly smeared-out surface charges (i.e., without possibilities for ion–site correlations) or whenever ion–site correlations are negligible, we have ξ^2^ = 1. Measuring the contact density, as on the right side of eq 1, and comparing it with 2π*l*_B_σ^2^ is hence an excellent way of quantifying the ion–site correlations. In general, ξ^2^ is small whenever the electrostatic coupling parameter is low and/or when the ion–site separation is large as compared to the neighboring site–site distance of the charged sites.

The hydronium activity *a*_H_3_O^+^_ is traditionally given in logarithmic units pH = −log_10_*a*_H_3_O^+^_ and the acid equilibrium constant p*K*_a_ = −log_10_*K*_a_ of the reaction −OH + H_2_O(l) *⇌* −O^–^ + H_3_O^+^(aq). In most cases, one assumes that the activity of the hydronium ion is close to its ideal/entropic part, that is, its concentration, by *a*_H_3_O^+^_ = [H_3_O^+^], where [H_3_O^+^] (usually abbreviated by [H^+^]) is the concentration in units of mol/L. *K*_a_ is then given in the same units. Our p*K*_a_ is assumed to be an intrinsic value, independent of salt types and their concentrations, as it is based on the individual species’ activities, which naturally include electrostatic contributions. This differs from the sometimes used “apparent” p*K*_a_^app^ values, which assume that activities are only given by the individual species concentrations, neglecting any energetic contribution to the activities. Such a quantity would naturally be heavily salt-dependent, and the use of such should be discouraged.

The ionization degree of the surface charge density α is given by the simple equilibrium relationship:
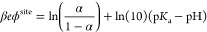
2where we assumed negatively charged (acid) surface groups (of the type −O^–^) and ϕ^site^ is the electrostatic potential on a surface group. The surface charge density is given by σ = −ασ^site^, where σ^site^ is the surface site density. Equation 2 can be rephrased into a two-state (deprotonated and neutral OH-groups) equilibrium equation, as
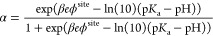
3

**Figure 1:**
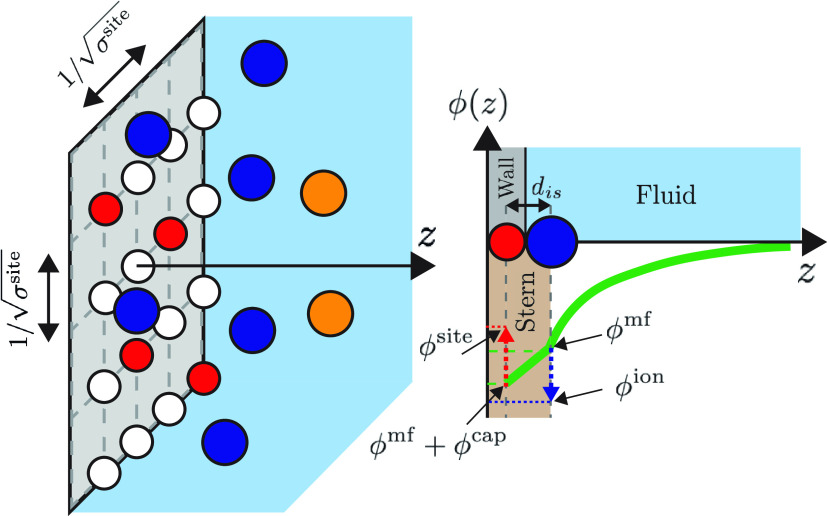
Left: Schematics of the system, showing the surface in gray with the sites as circles in either white (neutral) or red (negatively charged). Sites are arranged in a square lattice on the surface, with the lattice dimensions . Blue circles illustrate counterions (cations) and orange represent co-ions (anions). Right: Schematics of the electrostatic potential, ϕ(*z*), perpendicular to the surface, where *z* denotes the distance to the surface. The green curve illustrates how the mean-field plane-averaged electrostatic potential varies along *z*, including the Stern layer/capacitance linear increase from the closest approach of an ion’s charge to the charge of a site (given by *d*_is_). An ion at the surface experiences the mean-field potential ϕ^mf^ but also ion–ion correlations and excluded volume contributions that increase the electrostatic potential in absolute terms. The resulting average electrostatic potential on the ions at the surface, ϕ^ion^, is depicted in the figure. The plane-averaged mean-field electrostatic potential in the sites’ plane is given by ϕ^mf^ plus the capacitance term ϕ^cap^ (see figure). On top of that, the discrete sites experience site–site correlations, yielding a reduced potential in absolute terms. The resulting potential, ϕ^site^, is depicted in the sketch. Note that ϕ^mf^ and ϕ^mf^ + ϕ^cap^ both are plane-average mean-field potentials, whereas ϕ^ion^ and ϕ^site^ are localized potentials on the ions at the surface and sites, respectively.

We decompose the electrostatic potential on a salt ion at the surface as ϕ^ion^ = ϕ^mf^ + ϕ^ii^ + ϕ^ex^, where ϕ^mf^ is the mean-field plane-averaged electrostatic potential at the surface (see Figure 1), ϕ^ii^ is an electrostatic potential correction on the ion due to ion–ion correlations at the interface, and ϕ^ex^ is an excluded volume correction to the electrostatic potential due to the finite size of an ion. Similarly, one can decompose the site potential as ϕ^site^ = ϕ^mf^ + ϕ^cap^ + ϕ^ss^, where ϕ^cap^ is the capacitance contribution and ϕ^ss^ is a correction due to site–site correlations. This decomposition is based on the fact that there is no significant ion–site correlation, and hence the potentials of the ions and sites only couple via the mean-field potential ϕ^mf^ (see Figure 1) . The capacitance *C*_S_ is related to the minimum separation between ions and surface sites (also denoted as the Stern layer). This gives rise to a potential contribution as ϕ^cap^ = *e*σ/*C*_S_, where *C*_S_ = ε_0_ε_r_/*d*_is_, *d*_is_ is the closest separation, perpendicular to the surface, between the charges of an ion and a site, and ε_0_ε_r_ is the absolute dielectric permittivity. We approximate the site–site correlations as
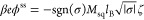
4where *M*_sq_ ≈ 1.949 is the Madelung constant for a square lattice.^18^ The choice is motivated by the site structure used in the MC simulations. This term corresponds to a 2D square lattice crystal energy where the charges are at a maximum distance away from each other on the lattice. The 0 < ζ < 1 parameter accounts for the fact that sites do not correlate perfectly for nonfully occupied lattices at room temperature, essentially incorporating the entropy effects of lattice gas. This parameter is one out of two unknowns in the current theory. However, as we will see later, it can be fine-tuned for one salt case and then kept constant for all other cases.

The ion–ion correlations are approximated via a first loop correction to the mean-field^19^ as β*e*ϕ^ii^ = −*c*_0_Ξ/*z*_s_, where *c*_0_ = (π/8 – 0.3104) ≈ 0.08230, and where Ξ is an average coupling parameter, and *z*_s_ is the average charge of the ions residing at the charged surface. However, the ion–ion correlation energy, assuming perfect neutralization, cannot exceed a corresponding Madelung energy, where all counterions are bound to the surface and form a square or hexagonal lattice. Assuming that the ions form a 2D hexagonal layer, being the lowest energy state of the two-dimensional single-species ionic crystal, the energy per charged site is given by  with *M*_hex_ ≈ 1.961.^18^ Note that the Madelung constants of a square, *M*_sq_ = 1.949, and hexagonal lattice, *M*_hex_ = 1.961, differ only by less than 1%; hence, an exact knowledge of which lattice is preferred is not essential for the current theory. To incorporate this upper energy limit, we use a modified ion–ion correlation:
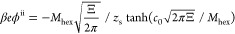
5having the right limits at both high and low Ξ’s. While the loop correction is derived for a salt-free system, we assume the correction is also approximately valid for a salt system in the vicinity of the surface as most of the ions there are counterions.

The average valency of the ions residing at the interface is given by
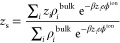
6

For highly charged surfaces, one obtains the valency of the counterion, but it is considerably lower for lower surface charges. In the limit of zero surface charge, the average valency equals zero, as it should. The motivation behind using the contact valencies is that the ion–ion correlations are the most important there (as the overall density is at its highest at the interface). Following refs (20) and (21), we define the average coupling parameter as Ξ = 2π*l*_B_^2^σ*z*_s_^3^.

The excluded volume correction to the electrostatic potential of the ions can be approximated by , where *d*_ii_ is the closest separation between two ions and is similar in spirit to the hole corrected Debye–Hückel theory.^22,23^ This term, linear in σ, resembles a parallel capacitor correction. While there might be a coupling/overlap between the ion–ion correlation and excluded volume, in this theory, we consider them independent of each other (see the Supporting Information for the effect of excluding this term).

The only undefined quantity is now the ϕ^mf^, but this quantity is redundant as it appears in both eqs 1 and 2. The surface charge density can be solved self-consistently. This can numerically be done by first assuming some values for ζ and ξ^2^ (typically to 0.75 and 1, respectively) and then guessing an initial value for σ. This guess provides us with values for ϕ^ex^ and ϕ^cap^. From eq 1 and given the salt type and its concentrations, we can then obtain a guess of ϕ^ion^, which can be used to determine *z*_s_ and Ξ, and hence ϕ^ii^. By eliminating ϕ^mf^, we find ϕ^site^ = (ϕ^ion^ – ϕ^ii^ – ϕ^ex^) + ϕ^cap^ + ϕ^ss^. For a given pH value, we use eq 2 to get the degree of ionization α, from which an updated guess of σ = −ασ^site^ is obtained. This procedure is then iterated until σ does not vary more between single iterations. Note that, in this procedure, it is very easy to “turn off” one or several of the terms by simply putting the corresponding potential(s) to zero.

## Results and Discussion

We study the exact same systems as in refs (2) and (10). To minimize the number of free parameters in our theory, we used the exact same microscopic parameters (i.e., site densities, p*K*_a_ values, etc.) as the corresponding Monte Carlo simulations.^10^ These parameters have been shown to successfully reproduce the experimental data of ref (2) when using Monte Carlo simulations and the primitive model. As our theory is based on the exact same model, the MC data serve as an excellent test of the current theory. The only further unknown parameters we have are ξ and ζ, where the latter is upper and lower bounded. The theory could equally be used to fit the experimental data. The above microscopic parameters would then again be unknown/free parameters within reasonable physical limits (with or without the help of other complementary experimental techniques), just like they were in the MC simulations.

From ref (10), we obtain *l*_B_ = 0.714 nm corresponding to pure water at room temperature, σ^site^ = 4.8 sites/nm^2^ (corresponding to a maximum surface charge density of −770 mC/m^2^), p*K*_a_ = 7.7, *d*_is_ = 0.35 nm, and *d*_ii_ = 0.4 nm. These values give *C*_S_ = 1.93 F/m^2^, which is lower than the value *C*_S_ = 2.6 F/m^2^ used in ref (10) to fit the surface complexation model with their MC results for simple 1:1 salts. However, combining the Stern capacitor with the “excluded volume” capacitor gives *C*_eff_ = (*C*_S_^–1^ + *C*_ex_^–1^)^–1^ = 2.67 F/m^2^, a value very close to the empirical fitted value of ref (10). This gives a theoretically sound explanation of the increased capacitance needed in mean-field theories to agree with MC results. Furthermore, two kinds of simulations were carried out in ref (10): a set of simulations with discrete surface site charges and the other with uniformly smeared-out surface site charges. We will compare our theory to both of these. For more information about the MC simulations, see ref (10).

**Figure 2:**
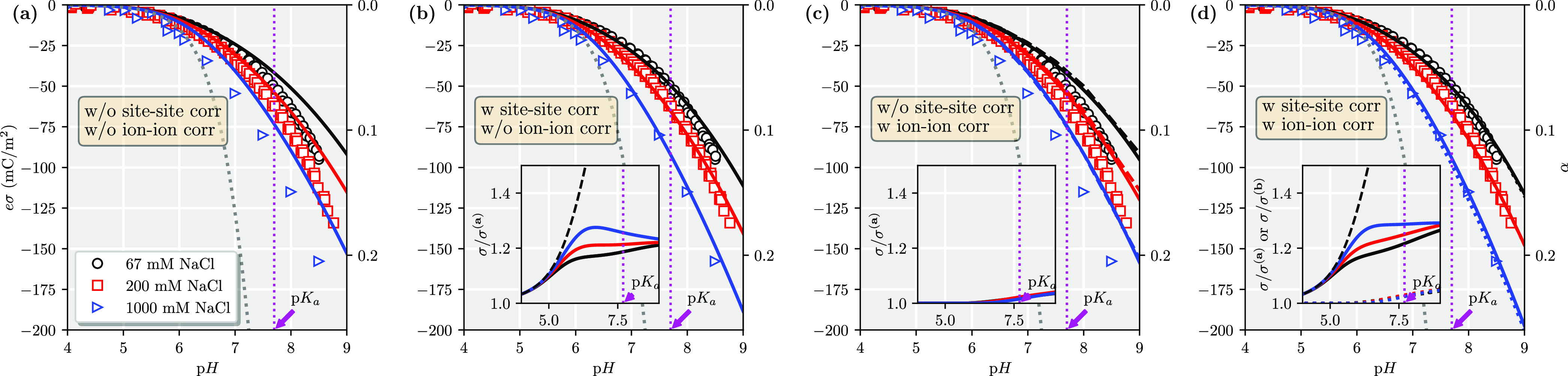
Comparison between the different levels of theory using ζ = 0.75 and ξ^2^ = 1: (a) simple capacitor, (b) with site–site correlations, (c) with ion–ion correlations, and (d) with both ion–ion and site–site correlations for a 1:1 salt at various concentrations. Markers are experimental data,^2^ solid lines are theory, and dashed/dotted lines are MC results,^10^ where dashed lines show MC results for smeared-out surfaces and dotted for discrete charges. A gray dashed line shows the Henderson–Hasselbach prediction. Insets show (solid lines) the relative increase as compared to (a) by including various terms. The inset of (d) also shows (dotted lines) the relative increase, including ion–ion correlations, as compared to having only site–site correlations included (i.e., as compared to (b)). The dashed line in the inset shows the prediction according to eq 7.

We start our analysis by assuming that ion–site correlations are negligible, setting ξ^2^ = 1. The solid lines in Figure 2 show the effect of accounting for ion–ion or/and site–site correlations or none to the surface ionization at various 1:1 salt concentrations. Best fits to the data were found using ζ = 0.75. Using ζ = 1 only slightly overestimates the surface ionization as compared to MC, and using ζ = 0 corresponds to having no site–site correlations (see Figure 2a and c). The largest ionization is found when both correlation terms have been included. As compared to the ionization profile without any correlations, we see that site–site correlations have the largest effect, increasing the ionization with ∼20% for pH > 6 (see inset) in all of the cases. Including only ion–ion correlations increases the relative ionization degree by ∼5%, and that at the highest pH-values. The combined effect of the two correlations raises the relative ionization by ∼20–30% for pH > 6.

We can compare our theory to MC simulations, which also (c) used uniformly smeared-out site charges and (d) accounted for site discreteness. Comparing our theory (solid lines) to their numerical data (dashed or dotted lines) shows an impeccable agreement, with lines being almost indistinguishable, using the same level of approximation (uniform vs discrete). In our theory, assuming that ions–site correlations are negligible (i.e., using ξ^2^ = 1), the only free parameter has been ζ. This parameter does, however, only mildly affect the ionization curves, as the case ζ = 1 shows (see Figure S1a). Overall, the theory with site–site and ion–ion correlations incorporated and MC simulations agrees well with the experimental data. Combining eqs 2 and 4, we find that the relative surface charge density increases by
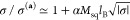
7at low surface ionizations when site–site correlations are included as compared to without (the latter denoted by σ^(**a**)^, and corresponding to the lines shown in Figure 2a) at the same salt and pH conditions. At lower pH values, the typical increase in surface charge densities is low and within the noise of the experimental data or MC results.

**Figure 3:**
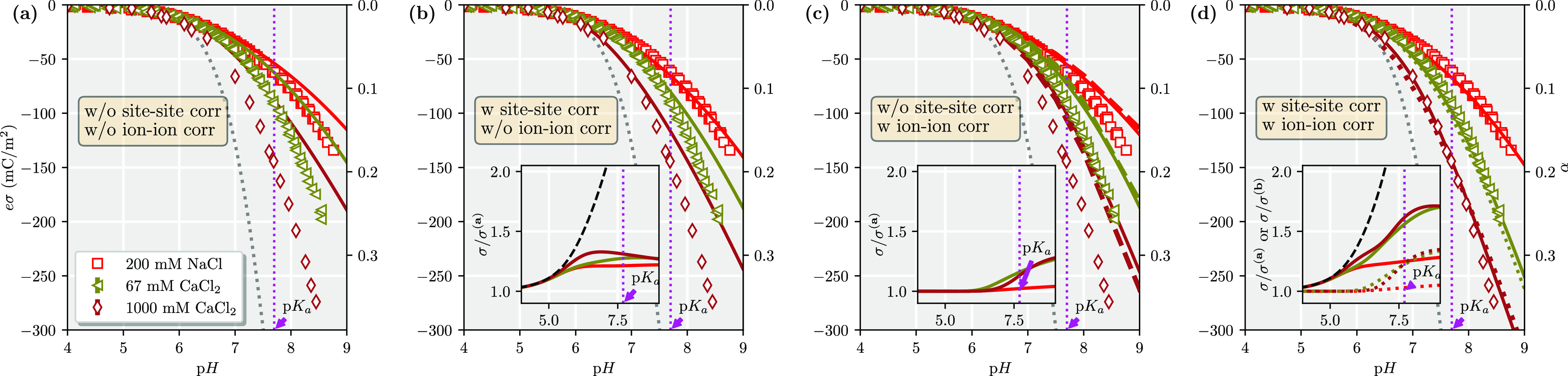
Comparison between the different levels of theory using ζ = 0.75 and ξ^2^ = 1: (a) simple capacitor, (b) with site–site correlations, (c) with ion–ion correlations, and (d) with both ion–ion and site–site correlations for a 2:1 salt at various concentrations (200 mM 1:1 salt is included as a reference). Markers are experimental data,^2^ solid lines are theory, and dashed lines are MC results,^10^ where the dashed lines show MC results for smeared-out surfaces and dotted for discrete charges. The gray dashed line shows the Henderson–Hasselbach prediction. Insets show (solid lines) the relative increase as compared to (a) by including various terms. The inset of (d) also shows (dotted lines) the relative increase, including ion–ion correlations, as compared to having only site–site correlations included (i.e., as compared to (b)). The dashed line in the inset shows the prediction according to eq 7.

We continue testing our theory for 2:1 salts. Again, we neglect any ion–site correlation, setting ξ^2^ = 1. Figure 3 compares our new approach, including various correlation terms, with experimental and MC results, using the same ζ-parameter as was fine-tuned for the 1:1 salt cases. Notice that the 67 mM CaCl_2_ and 200 mM NaCl have the same Debye screening length, and, hence, the linearized Poisson–Boltzmann (i.e., Debye–Hückel) would predict the same titration curve for the two salt cases. The difference between the two corresponding theory lines in Figure 3a, without any correlations, is therefore due to the nonlinearity of the Poisson–Boltzmann equation, which starts to be significant at high surface potentials and high ion valencies. Figure 3b shows that predictions, as compared to the experimental data, can be improved by including only the site–site correlations up to a surface charge density of σ ≈ 100 mC/m^2^ for the 2:1 salt. Including also ion–ion correlation effects into the theory improves the accuracy for higher surface charge densities, as seen from Figure 3d, where theory, MC simulations, and experimental data all give the same curves for a given salt concentration. Comparing Figure 3a–d, we see that site–site correlations are still the major contributor to an increased surface charge density, with a relative increase by ∼20–30% for pH > 6. At pH > 7.5, when the surface charge density is high, ion–ion correlations give a further ∼20–30% relative increase. This finding contrasts with the 1:1 salt cases, where we found that ion–ion correlations are largely negligible for all of the studied surface charge densities. As divalent ions now act as counterions to the surface, the corresponding coupling parameter Ξ ≈ σ*z*_s_^3^ is roughly a factor of 8 bigger than the 1:1 salt case at a comparable surface charge density, explaining the larger importance of ion–ion correlations in the former but not the latter. Their combined effect gives an increase of up to 75% at the highest pH values, that is, larger than the sum of the individual contributions. Hence, the two correlations work in a synergetic manner.

Again, we can compare to MC simulations with the same level of approximation the (c) uniformly charged surfaces and (d) discrete surface sites for 2:1 salts and find excellent agreement. This indicates that each correlation term is well approximated.

So what about possible ion–site correlations? Are those negligible? To answer this question, we need to know the ξ^2^ values. ξ^2^ depends nonlinearly on both Ξ and the relative site–ion separation , but increases with increasing Ξ and decreases with increasing . For the 1000 mM 1:1 salt and pH = 9, we have a coupling parameter of Ξ ≃ 4 and . Comparing with the results of ref (16) (see Figure 2a in that reference), we see that Ξ = 10 (and  gives ξ^2^ = 1.1), which should indicate that we have ξ^2^ = 1 in our case based on both our lower coupling parameter and our higher relative ion–site separation. Lowering Ξ to ≃1 in our case gives us , as . This still gives ξ^2^ = 1 as Moreira and Netz found ξ^2^ = 1 for Ξ = 1 and  (see Figure 2b of ref (16)). Hence, for the studied 1:1 salt cases, ξ^2^ can be considered to be equal to 1, which was also assumed in the above analysis, even though there is a small possibility that an open system behaves slightly differently (ref (16) studied the salt-free case).

For 1000 mM 2:1 salt, we instead have Ξ ≃ 50 and a relative ion–site distance  at pH = 9. Comparing with the results of ref (16) (see Figure 3 of ref (16)), we determined that a value of ξ^2^ = 3 and Ξ ≃ 50 should occur at . Increasing the relative ion–site separation will naturally decrease ξ^2^. For Ξ = 10 (see Figure 2a of ref (16)), ξ^2^ reduces from 2.9 to 1.1 as the relative ion–site separation is doubled from 0.12 to 0.24. Hence, even for the 2:1 salt cases, we can assume that ξ^2^ is close to 1. If we instead use ξ^2^ as a free parameter (with ζ = 1) for the 1000 mM 2:1 salt case, we can reproduce the MC simulations’ data by using ξ^2^ = 3 (see Figure S2). This is, however, a value much higher than ref (16) would support. Hence, also for the 2:1 salt cases, it seems reasonable to assume ξ^2^ = 1 for the given microscopic parameters. Table 1 summarizes the values used in the above arguments. When using ξ^2^ > 1, we have neglected that including ion–site correlations would most likely reduce both the ion–ion and the site–site correlations. Further improvements of the current theory naturally need to address the cases where ion–site correlations could be important.

**Table 1:** Summary of the Measured and Obtained ξ^2^ Values for the Various Pairs of Ξ and  Parameters as Discussed in the Text^a^

case	Ξ		ξ^2^	source
salt free	1	0.12	1	Moreira and Netz^16^
salt free	10	0.12	2.9	Moreira and Netz^16^
salt free	10	0.24	1.1	Moreira and Netz^16^
salt free	50	0.2	3	Moreira and Netz^16^
1000 mM 1:1	1	0.2	≃1	this work
1000 mM 1:1	4	0.4	≃1	this work
1000 mM 2:1	50	0.5	≃1	this work

a Measured ξ^2^ values are all from ref (16), while the ones listed as [this work] are estimates based on the measured values and their behaviors. The table only includes the highest studied values for each salt case (1000 mM and at pH = 9).

Note that, in the above arguments, we used our average coupling parameter. However, whenever the average coupling parameter is high, this is near-equal to the standard counterion-only coupling parameter. This is also the regime where ion–site correlations could be important. In fact, the average coupling parameter essentially only regularizes the low surface charge densities at low pH values, being the same as the standard coupling parameter at higher surface charge densities, corresponding to high pH values.

Are then ion–site correlations never important? No, they become important as one increases the valency of the counterions (from divalent to trivalent) or reduces the minimum separation between the site charges and counterions. This can be easily done in MC simulations by lowering *d*_is_, but it would simultaneously also affect the capacitance term. Hence, the outcome is not straightforward to predict. A lowered *d*_is_ value would correspond to neglecting the ions’ and sites’ hydration layers, something that is not standardly done within the primitive model.

Labbez et al.^10^ also explored the effect of surface charge density as two surfaces approach each other and found that the curve was nonmonotonic for 2:1 salts and high pH values. For finite distances, one has a finite pressure β*P*. According to the contact theorem,^12−15^ this nonzero pressure leads to a correction term on the right side of eq 1 as
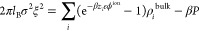
8

This equation is valid for all three standard boundary conditions (acting on the surfaces): constant charge (CC), constant potential (CP), and charge regulating (CR). CC corresponds to an insulator, CC to a conducting surface (electrode), and CR to a surface with ionizable groups. The difference between them is what is kept constant and what is allowed to vary as a function of surface-to-surface separation. For the CC condition, σ is constant, for the CP condition ϕ^site^, and for the CR condition neither are constant, and σ and ϕ^site^ instead related to each other via eqs 2 and 8. The charge regulating (CR) condition is typically between the limiting conditions of constant charge (CC) and constant potential (CP).^24^ By definition, the CC condition yields constant surface charge densities for all separations. However, the surface charge densities for the CP condition would depend on the pressure. For a 1:1 salt, the pressure increases monotonically as the separation decreases; hence, the surface charge density decreases monotonically as a function of decreasing separation. However, for a 2:1 salt, there exist regimes where the pressure is attractive due to ion–ion correlations.^25^ At these separations, the surface charge density would instead increase, according to eq 8. CR being a mixture of CC and CP, one quickly sees that the CR condition has the same qualitative behavior as the CP condition; that is, the surface charge densities follow the pressure curve trend of the CP condition. Hence, we can understand the increasing surface charge densities as a function of decreasing separation for 2:1 salts and high pH values due to the presence of attractive ion–ion correlation-mediated surface–surface forces in line with the conclusions of ref (10).

## Conclusion

We have presented a new and simple theory for the titration behavior of charged planar surfaces, including site–site and ion–ion correlations. Our approach is in excellent agreement as compared both to experiments and to Monte Carlo simulations. The results show that site–site correlations always are at play, being one of the dominant correction terms. In contrast, the ion–ion correlations are first significant for divalent salts and high surface charge densities, for example, high effective coupling strengths. Including both site–site and ion–ion correlations increases the ionization degree by 30% for 1:1 salts and by 75% for 2:1 salts. We find that ion–site correlations can largely be neglected, at least for the corresponding studied MC cases.

This work generalizes the ion–ion correlation corrections approach for salts, relying on an effective coupling parameter, different and complementary to the dressed ion approach.^26−29^ Further work will include excluded volume effects, ion-specific effects, cases where ion–site correlations are important, and detailed investigations of the density profiles close to the surfaces.^11,30^

## Supporting Information Available

Additional figures where various parameters have been varied (ζ, ξ^2^, and ϕ^ex^) (PDF)
