# Supplementary material for: Ion and Site Correlations of Charge Regulating Surfaces: A Simple and Accurate Theory
Source: Langmuir. 2023 May 24;39(22):7642–7. doi: 10.1021/acs.langmuir.3c00316 (PMC10249413; doi:10.1021/acs.langmuir.3c00316)
Supplement: Supplementary file 1 — la3c00316_si_001.pdf [file la3c00316_si_001.pdf]

Supplementary Information: Ion and Site Correlations of Charge Regulating Surfaces: A Simple and Accurate Theory

Martin Trulsson*

Computational Chemistry, Lund University, SE-221 00 Lund, Sweden

E-mail: martin.trulsson@compchem.lu.se

Fig. S1 shows the effect of using $\zeta = 1$, including ion-ion correlation and ion-site correlations, on the titration behaviour as a function of pH at various concentrations of **(a)** 1:1 salts and **(b)** 2:1 salts. There is a slight increase in surface charge density at the same conditions (*i.e.*, salt concentration and pH), leading to a slightly worse agreement with the Monte Carlo results. However, the error is less than $\sim$5%, found at the highest pH values, and given the approximative theory, this is still a tolerable error.

Fig. S2 shows the effect of having $\zeta = 1$ and $\xi^2 = 3$ for a couple of salts. While the 1000 mM 2:1 salt gives a good agreement with MC simulations (and experimental data), the agreement is only fair for 67 mM 2:1 salt and becomes worse for a 200 mM 1:1 salt.

Fig. S3 shows the effect of neglecting the ϕ^{ex} term, using $\zeta = 1$. All the curves show a weaker ionisation for the same salt, concentration, and pH. Lowering ζ or increasing ξ^2 will further decrease the ionisation, *i.e.*, is not a viable approach.

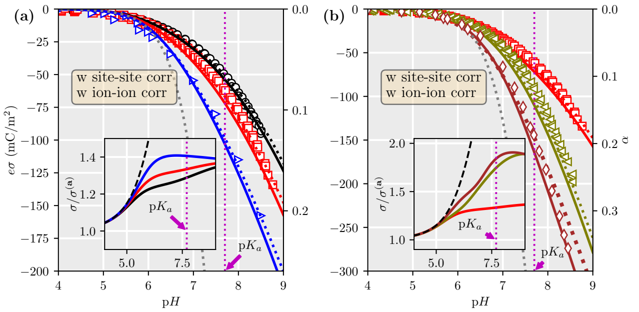

Figure S1: **(a)** Same as Figure 2**(d)** but with $\zeta = 1$. **(b)** Same as Figure 3**(d)** but with $\zeta = 1$.

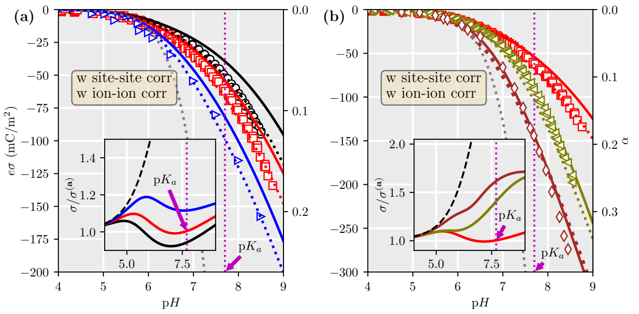

Figure S2: **(a)** Same as Fig. S1**(a)** but with $\xi^2 = 3$, **(b)** same as Fig. S1**(b)** but with $\xi^2 = 3$.

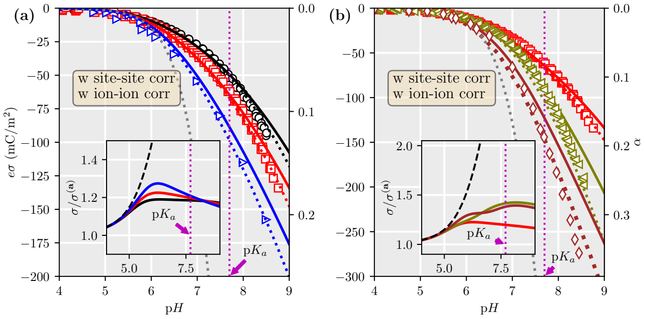

Figure S3: **(a)** Same as Fig. S1**(a)** but without ϕ^{ex}, **(b)** same as Fig. S1**(b)** but without ϕ^{ex}.
